# Unveiling of phytochemicals, antioxidants, cytotoxic effects and anthelmintic potency of green tea (*Camellia sinensis*) beverage against albendazole resistant *Hamonchus contortus*

**DOI:** 10.1038/s41598-026-42739-x

**Published:** 2026-03-25

**Authors:** Noha M. F. Hassan, Bassma S. M. Elsawy, Mohamed A. Helal, Marwa H. Mahmoud, Nadia M. T. Abu El-Ezz, Hatem A. Shalaby

**Affiliations:** 1 Parasitology and Animal Diseases Department, Veterinary Research Institute, National Research Centre, P.O. 12622, Dokki, Egypt; 2 Food technology Department, Food Industries Institute, National Research Centre, P.O. 12622, Dokki, Egypt

**Keywords:** Haemonchosis, Albendazole resistance, Sheep, Green tea catechines, Anthelmintic activity, Biochemistry, Biotechnology, Drug discovery, Microbiology, Plant sciences

## Abstract

*Haemonchus contortus* remains the main multidrug resistant strongyle threatened livestock productivity. The phytochemical components of green tea beverage (GT) might provide an alternative and sustainable anthelmintic effect. Therefore, this study aimed to evaluate the anthelmintic effect of GT beverage against albendazole resistant *H. contortus* from naturally infected sheep. Albendazole resistance was detected through egg hatch inhibition assay. Assessment of GT phytochemicals, antioxidant ability, anthelmintic effect and cytotoxicity MTT (dimethylthiazol-diphenyltetrazolium bromide) colorimetric assay were achieved. The GT was of high Total Phenolic Content; 555.32 mg gallic acid equivalents /g. Remarkable GT antioxidant qualities were noted where, 1,1-diphenyl-2-picrylhydrazyl (DPPH) radical scavenging activity (85.14%), DPPH antioxidant activity as 907.57 mg vitamin C/g equivalents, and Ferric Reducing Antioxidant Power (8991.04 µg Trolox/g). High Performance Liquid Chromatography showed rich profile of phenolics mainly catechine (70,190.8 µg/g). A potent anthelmintic activity of the GT was noted at 50 mg/mL; complete inhibition of egg hatchability (LC_50_; 0.144 mg/mL), significant larval motility inhibition (LC_50_; 0.127 mg/mL) and severe structural deformity on *H. contortus* eggs and larvae. Additionally, 100% worm motility inhibition and mortality index 1 were recorded at 400 mg/mL in 2 h incubation (LC_50_; 13.387 mg/mL). The light and electron scanning microscopy of the treated adult proved distortion in the muscular layer of the cuticle wall with severe degenerative changes. The GT maintained viability of the BJ1 cell lines without cytotoxic effect at (0.78 to 200 µg/mL) concentrations. Overall, green tea beverage could be offered as safe and potent alternative anthelmintic to combat the albendazole-resistance isolates of *H. contortus*.

## Introduction

Sheep farming is one of the crucial sectors for food security purposes all over the world^1^. *Haemonchus contortus* is the hazard gastrointestinal strongyles that parasitize the abomasum of various livestock in tropical and subtropical regions^2–4^. It is a highly pathogenic hematophagous nematode causing anemia, hemorrhagic gastroenteritis, appetite depression, hypoprotenemia, and in severe infection, it could prompt sudden death or chronic emaciation^5,6^.

Over decades, the control of haemonchosis has relied on the utilization of synthetic anthelmintics^7^. Consequently, the risk of environmental outbreak of the drug residues increased, along with development and mass spreading of resistant populations of helminthes associated with the mal efficacy of anthelmintics^8^. Multiple studies recognized *H. contortus* as the resistant strongyle to all anthelmintic drugs, which adversely impacted on livestock production and exaggerated economic losses^9^.

Exploring the anthelmintic features of plants containing bioactive metabolites is a promising alternative green approach^10–12^.

Green tea: *Camellia sinensis* L. *Kuntze* (*Theaceae*), is a common worldwide beverage exhibited with a broad spectrum of biological activities. The most precious green tea components are catechins, a group of polyphenols of pharmaceutical significance. Green tea acts as an anti-inflammatory agent^13^, anti-cancer agent^14^, antihypercholesterolemic^15^, anti-microbial and antiviral^16,17^. It can enhance metabolic biomarkers^18^ and lower body weight^19^, and it revealed a beneficial effect on cardiovascular diseases^20^. The polyphenols antioxidant activities are able to scavenge free radicals and reactive oxygen species, maximize the effectiveness of green tea in improving health condition^21,22^.

Anthelmintic activity of green tea has been tested on *Plasmodium berghei*^23^, *Leishamania amazonenesis*^24^ and *Acanthamoebic castellanii*^25^. Moreover, Zhong et al.^26^, and Ramdani et al.^27^, mentioned the ability of dietary green tea supplementations to reduce strongyle worms. Several approaches used to assess anti-parasitic therapeutic potency of plant bioactive compounds, including in vitro anthelmintic assays^28^, parasitic ultrastructural deformity determinants^29^ and cell culture assay for toxicity screening^30^ are needed to understand basic mechanisms, that simulates an in vivo test or clinical use.

The direct anti-nematodal effect of green tea beverage, and the proper concentrations which can eliminate this obstinate parasite without adverse effect on the host are still not clear yet. Therefore, the objective of the present study was to investigate phytochemical composition, antioxidant ability and the potential anthelmintic effect of the green tea cultivated in Egypt, based on the green tea beverage bioactive polyphenolic constituents, against the albendazole resistant *H. contortus* isolate eggs, larvae and adults as a green candidate for combating haemoncosis in additions determination of the toxicity of the prepared GT via detecting the possible cell viability.

## Materials and methods

This study has been approved by institutional guidelines of the National Research Centre’s Animal Research Committee: Medical Research Ethics Committee (the approval number: 13050425). All methods were performed in accordance with the relevant guidelines and regulations.

### Plant

#### Extraction

A permission was taken to collect green tea leaves from National Research Center farm, in Nubaria, Egypt. The plant was identified by botanists in The National Research Centre. The leaves were cleaned manually to remove all foreign materials and dried in the shade. Then, it was blended to powder (0.5 mm) form of a high-speed blender (Braun KMM 30 mill, type 3045, CombiMax, Germany). Green tea leaves powder (10 g) was extracted with 100 mL of ethanol 70% in an ultrasonic device (200 W, 59 kHz, Shanghai Kudos Sonication Machine Company Ltd., China) for 60 min at room temperature. The mixture was then filtered through Whatman no. 4 filter paper, and the filtrate was evaporated under reduced pressure at 30 ˚C until its volume was about 40 mL. The final volume of the extract was made to 50 mL with the extraction solvent and then it was taken out for the analysis^31^.

#### Determination of total phenolic compounds

The total phenolics in the GT extract were measured utilizing the Folin–Ciocalteu method according to Ramful et al.^32^, which was modified by Mahmoud et al.^33^. After adding 3.5 mL of distilled water and 0.25 mL of Folin–Ciocalteu reagent (Merck) to 0.25 mL of diluted extract (ethanolic extract 70%). In place of plant extract, a blank was made with 0.25 mL of 80% methanol. Three minutes later, one milliliter of 20% sodium carbonate was added. The contents of the tube were vortexed and then incubated in a water bath at 40 °C for 40 min. In comparison to the blank standard, the absorbance of the blue coloring that developed was measured at 685 nm. Calculations of total phenolics were made using the gallic acid standard curve (concentration range: 0–12 µg/mL). Results were expressed as mg of gallic acid per 100 g of plant material.

#### Antioxidant activities

##### DPPH radical scavenging activity

To estimate the impact of extracts on 1,1-diphenyl-2-picrylhydrazyl (DPPH) free radicals, Aboelsoued et al.^34^ provided a method. The absorbance was measured using a spectrophotometer at 517 nm. Ethanol was used as the control in place of the sample. This Eq. (1) was used to determine the DPPH scavenging capacity:

1 $${\text{Scavenging activity }}\left( \% \right){\text{ }} = {\text{ A}}_{{\mathrm{c}}} {-}{\text{ A}}_{{\mathrm{s}}} /{\text{ A}}_{{\mathrm{c}}} \times {\text{ 1}}00{\text{ }}$$

Equation (1) Determination of DPPH scavenging capacity where Ac and As are the absorbance’s at 517 nm of the control and sample, respectively. L-Ascorbic acid solutions as standards were also analyzed by DPPH method. The total antioxidant values of samples were expressed as mg g^–1^ DW L-ascorbic acid equivalent antioxidant capacity (VCEAC)^35^.

#### Determination of Ferric Reducing Power (FRAP) assay

The capacity of phenolics to convert Fe³⁺ to Fe²⁺ is the basis for the FRAP test^36^. 10 mM TPTZ, 20 mM ferric chloride (10:01:01, v/v/v), and 0.1 M acetate buffer (pH 3.6) were combined to create the FRAP reagent. There were 150 µl of reagent and 20 µl of the previously diluted extract. A microplate spectrophotometer set to 593 nm was used to measure the absorbance. The results were reported as Trolox equivalents in µmoles per 100 g of FW sample.

#### Determination of individual polyphenols by HPLC

Agilent 1260 series equipment was used for HPLC analysis. Using a Zorbax Eclipse Plus C8 column (4.6 mm x 250 mm i.d., 5 μm), the separation was performed. The mobile phase was made up of 0.9 mL/min of water (A) and 0.05% trifluoroacetic acid in acetonitrile (B). The following is the sequential linear gradient programming of the mobile phase: 0–1 min (82% A); 0–11 min (75% A); 11–18 min (60% A); 18–22 min (82% A); 22–24 min (82% A). 280 nm was the monitoring wavelength for the multi-wavelength detector. Five microliters (µl) were the injection volume for every sample solution. At 40 °C, the column temperature kept constant.

### Detection of albendazole resistance

The study conducted on a private farm contained an albendazole resistant *H. contortus* infection among 150 balady sheep about 6 months to 3 years age, 25–75 kg weight, of different sex, located in Nahya, Giza governorate, Egypt. Animals suffered from repeated helminthes infection, illness with soft feces, weakness, weight loss and pale mucous membranes were noted, despite repeated oral administration of 2.5% albendazole, every 6 months where, the appropriate dose of 2.5% albendazole was determined according to the weight of each animal. Green forages, hay and dry ration were provided to animals with free access of tap water. Animals have not taken any anthelmintics for the last three months of the experiment. Egg hatch test is used to test albendazole resistance using Strongyle eggs collected from sheep. Then, the animals’ slaughtering has been humanely done at Nahya slaughterhouse, Giza province Egypt under the veterinarians’ authority. After that, *H. contortus* adult worms were utilized for obtaining monospecific resistant *H. contortus* donor. The farm owner had agreed to participate in this study.

#### Collection of strongyle eggs

Fecal samples were collected from each sheep and submitted to coprological examination utilizing sedimentation floatation test^37^. Strongyle eggs were assembled from positive fecal samples^28^. Briefly, fresh fecal samples were collected, homogenized utilizing a glass rod, sieved using 250, 100, and 25 μm sieves in a salt-saturated solution, and centrifuged at 2000 rpm for 20 min. The suspension of eggs was washed with distilled water three times to get rid of the excess of salt.

#### Egg hatch inhibition test

The egg hatch inhibition test was conducted as described by von Samson-Himmelstjerna et al.^38^. Stock solution of 400 µL of albendazole^®^ Pharma Swede, Egypt 2.5% was prepared, and diluted with 9.6 mL of Dimethyl sulphoxide (DMSO) 20%. Different concentrations (25 to 0.012 µg/mL) were prepared by 2-fold serial dilutions, using distilled water (DW) in a 24-well plate. The diluents were used as the control. One hundred strongyle eggs in 10 µL of DW were provided to each well. The test was done in triples. After that, the 24-well plate was tightly sealed using aluminum foil, to prevent dryness, and maintained at 27 °C for 48 h. To stop development of the eggs, about 10 µL of Lugol’s iodine were put per well. The number of unhatched eggs and first-stage larvae (L_1_) in each well were recorded utilizing inverted microscope (Olympus, CKX53, Japan) at 10× magnification. Mean hatch inhibition was calculated and lethal concentration 50 (LC_50_) was determined according to Coles et al.^28^.

#### Mono-specific albendazole resistant *H. contortus* eggs and larvae donors

Upon the result of the previous experiment, fecal samples were collected from each infected sheep with albendazole resistant strongyle group, to separately apply fecal culture. Larval identification was done according to Urquhart^39^. Strongyle infected sheep, with high percentage of resistant *H. contortus* larvae were selected and humanely slaughtered at Nahia, Giza province Slaughterhouse, to collect resistant *H. contortus* worm. The collected adult *H. contortus* females were incubated at 27 ºC for 3 h to permit ovi-deposition. Then, *H. contortus* eggs culture was performed to obtain the third stage larvae infective^6^. One free-parasite lamb 5 months age, 20 kg weight was parasitologically examined, kept for 4 weeks in a separate pen. Just before the experimental infection, another coprological investigation was applied to ensure that there was no infection. Then, the lamb was exposed to an oral dosing of 5000 infective resistant *H. contortus* larvae in 10 mL DW and kept for 21 days. Fecal samples were used for recovery of resistant *H. contortus* eggs. Copro- culture was done for collection and identification of larvae by the larvoscopic Baermann technique. The infected lamb was humanely slaughtered at Nahya, Giza province abattoir, to collect *H. contortus* adult worms whereas, they were used for adult motility test and light and electron microscopy scanning.

### Anthelmintic efficacy of GT

#### Egg hatching inhibition

The egg hatching inhibition (EHI) assay was conducted^28^. Stock solution of GT 1 mg/mL DW was used, by adding 800 µL of the stock solution to 1200µLof DW. Eleven concentrations of the GT (50 to 0.048 mg/mL) were used. One hundred *H. contortus* albendazole resistant eggs in 20 µL of DW were pipetted into each well of a 24-well plate. Untreated eggs in DW served as negative control. The test was done independently three times and in three technical replicates. Following 48 h of incubation, a drop of 5% Lugol’s iodine solution was added for stopping egg hatching. Unhatched eggs and larvae were recorded using light microscopy examination ×10.

#### Larval motility inhibition test

Larval motility inhibition (LMI) test was applied according to Hassan et al.^10^. The *H. contortus* albendazole resistant larvae were harvested from coproculture. The larva was subjected to several washes by DW, saline, then centrifugation was done for 5 min at 700×g and washed three times with PBS containing streptomycin and penicillin at concentration of 4% and only active motile larvae were utilized. In a 24-well plate, an equal number of freshly collected L_3_ suspensions (100 larvae/200 µL) were exposed to individual GT at different concentrations (50 to 0.048 mg/mL). Untreated larvae in DW served as negative control. The plate was incubated at 27 °C for 24 h. The number of mobile and immobile larvae were counted using light microscope^40^. The larvae were being considered motile when they exhibit a mild mobility accelerated by plate agitation and exposure to a light source. The assay was performed independently three times and in three technical replicates. Results were expressed as percentage inhibition of larval motility of tested wells compared to control wells.

#### The adult motility inhibition

The adult motility inhibition (AMI) test was applied as illustrated by Santos et al.^40^. The albendazole resistant adult *H. contortus* worms were collected from the slaughtered sheep abomasa. They were subjected to several washes by DW and saline. Twenty adult worms were treated with different concentrations of GT (400 to 3.125 mg/mL in DW). The control adults were incubated in DW. The test was done independently three times, with three technical replicates for each treatment. The worms were observed at 2, 4, 6 and 24 h intervals, at 37 °C for 24 h (5% CO_2_) and the number of active motile and immotile worms was recorded for each concentration. The Worm motility inhibition % (WMI) and Immobility Index (%) post 24 h GT exposure was estimated in accordance with the following Eq. (2):

2 $$\begin{gathered} {\text{Worm motility inhibition \% = }}\begin{array}{*{20}c} {{\text{Count of the motile worm}}} \\ {{\text{in the control untreated - count of the motile worm in the treaded}}} \\ {{\text{Count of the motile worm in the treaded}}} \\ \end{array} {\text{ /}}\begin{array}{*{20}c} {{\text{Count of the}}} \\ {{\text{motile worm}}} \\ {{\text{in the control untreated}}} \\ \end{array} {\text{ }} \times {\text{ 100}}{\mathrm{.}} \hfill \\ {\text{Immobility Index (\% ) = No}}{\text{. of immobile worms/Total No}}{\text{. of worms }} \hfill \\ \end{gathered}$$

#### Alterations of adult worm cuticles

Light and Scanning electron microscopy were used for studying the changes in the cuticle of twenty resistant adult isolate post exposure to GT. Ten *H. contortus* adults were washed utilizing DW then incubated for 6 h, and 24 h at a concentration of 400 mg/mL GT. The worms were moved formalin 10%, dissected, fixed and processed for light microscopy examination^41^. The mid-body wall was photographed utilizing an Olympus CX41 microscope. For further investigation of ultrastructural changes in cuticle wall, scanning electron microscopy was carried out for 10 worms 24 h GT post exposure where, the washed treated adults were fixed in 4% glutaraldehyde/0.1 M for 24 h and followed the method of Martínez-Ortíz-de-Montellano et al.^42^.

### The cytotoxic activity of the GT

The cytotoxic activity test was detected on normal fibroblast cell line (BJ1) depending upon cell viability assessment by the mitochondrial dependent reduction of yellow MTT (3-(4, 5-dimethylthiazol-2-yl)−2, 5- diphenyl tetrazolium bromide) to purple formazan^43^. A Laminar flow cabinet biosafety class II level (Baker, SG403INT, Sanford, ME, USA) was utilized under a sterile condition. Cells were put in DMEM-F12 medium, antibiotic-antimycotic mixture of 1%; (10,000U/mL Potassium Penicillin, 10,000 µg/mL Streptomycin Sulfate and 25 µg/mL Amphotericin B) and 1% L-glutamine at 37 °C and 5% CO_2_. In fresh complete growth medium, cells were cultured for 10 days, after that the cells seeded (10 × 10^3^ cells/well) in 96-well microtiter plates for 24 h, at 37 °C under 5% CO_2_ utilizing a water jacketed CO_2_ incubator (Sheldon, TC2323, Cornelius, OR, USA). Media was pipetted, fresh medium (without serum) was added, and cells were incubated either alone (control negative) or with varies concentrations of GT to provide a concentration of (200-100-50-25-12.5-6.25.5.25-3.125-0.78.125.78 and 1.56 µg/mL). Following two days of incubation, medium was aspirated, 40ul MTT salt (2.5 µg/mL) were supplied to each well and kept for further 4 h. For stopping the reaction and dissolution of the formed crystals, 200µL of 10% Sodium dodecyl sulphate in deionized water was used per well and maintained 24 h at 37 °C. DOX were utilized as positive control at 100 µg/mL which cause complete death of cells 100% lethality^44^. Measurement of the absorbance at 595 nm was done through a microplate multi-well reader (Bio-Rad Laboratories Inc., model 3350, Hercules, California, USA), and a reference wavelength of 620 nm. The vehicle utilized for dissolving GT was DMSO and its final concentration on the cells was less than 0.2%. The alteration percentage in viability of cells was assigned in accordance with the following Eq. (3):

3 $${\text{Cell viability }}\% {\text{ }} = {\text{ }}({\text{Absorbance of extract}}/{\text{Absorbance of negative control}}) - 1){\text{ }} \times {\text{ }}100$$

### Statistical analysis

The prevalence was presented as a percentage of the number of animals infected in the total number of animals examined. Data were summarized by descriptive statistics for the overall prevalence in sheep. Data obtained from EHA, LMA and AMI were analyzed by ONE Way ANOVA, Duncan’s multiple range tests and SPSS (version 16) computer program. The p value < 0.05 was considered significant. A statistical significance for MTT assay was tested between samples and negative control (cells with vehicle) using independent t-test by SPSS 11 program. A probit analysis was carried out for IC_50_ and IC_90_ determination using SPSS 11 program.

## Results

### Characterization of plant bioactive compounds

#### Total phenolic compounds (TPC)

The TPC value was 555.32 mg gallic acid equivalents (GAE)/g sample, demonstrated the high polyphenolic component content of green tea.

#### Antioxidant activities of extracted bioactive compounds

The percentage of DPPH Radical Scavenging Activity value was 85.14%. Green tea extract demonstrated an exceptionally strong capacity to neutralize the free radicals.

#### Antioxidant activity as mg equivalents of vitamin C/g using DPPH

The equivalent of antioxidant as vit. C was 907.57 mg Vit C/g for the DPPH test expressed in vitamin C equivalents confirms the potency of green tea’s antioxidant activity.

#### Antioxidant activity using FRAP (Trolox equivalents/g)

With FRAP value was 8991.04 µg Trolox/g, green tea’s potent lowering ability was demonstrated. The ability to convert Fe³ to Fe², which is correlated with electron-donating antioxidant activity, is measured by this test.

#### Characterization of phenolic compounds by HPLC

**Fig. 1:**
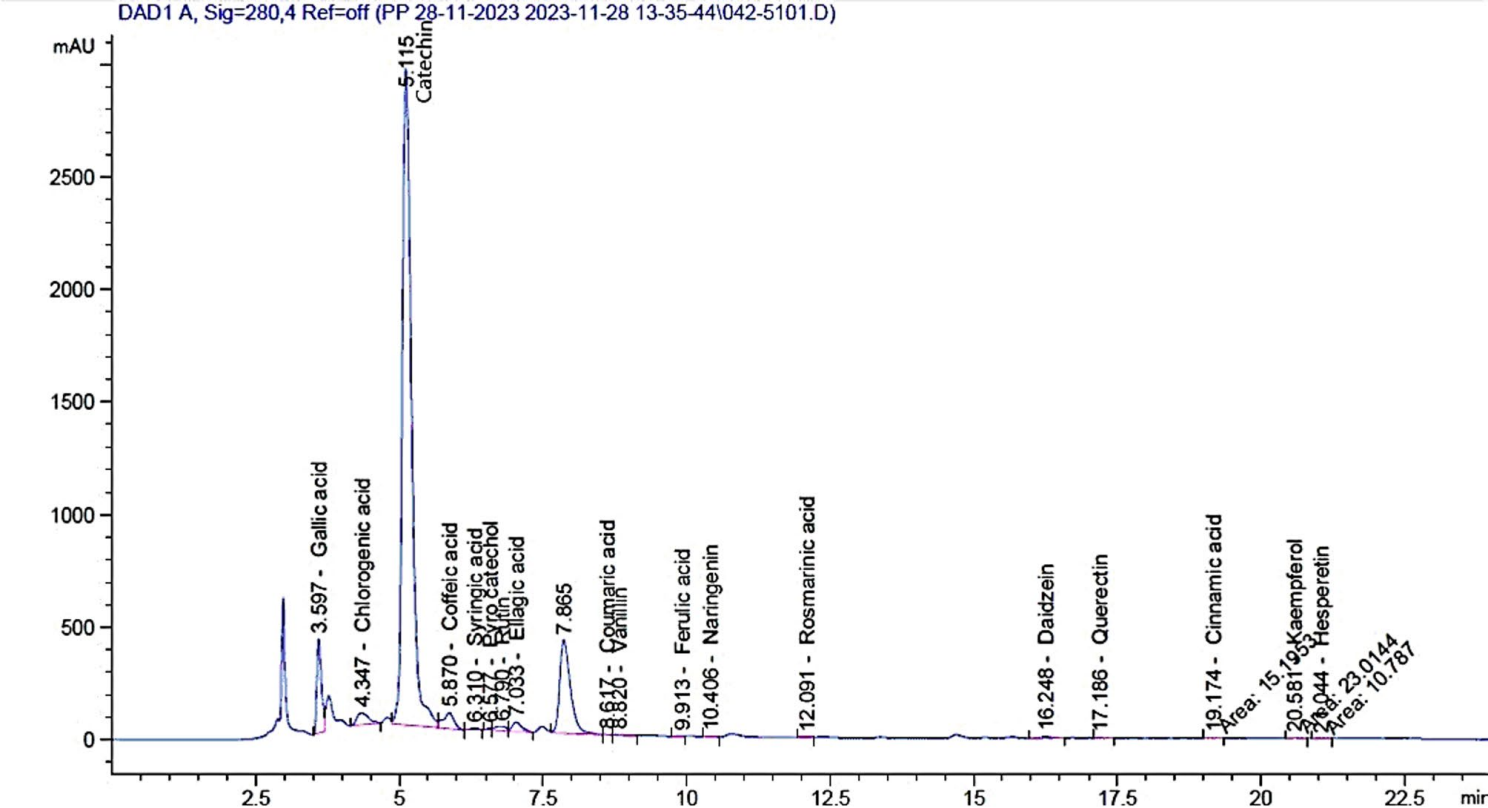
The chromatogram of the green tea leaves beverage.

The HPLC study of green tea revealed a rich and diverse profile of phenolic compounds, but with significant variance in their quantities (Table 1; Fig. 1). Green tea contains 70,190.8 µg/g of catechin, one of the primary phenols. Catechin is the most common phenolic component found in green tea. This important flavan-3-ol belongs to the catechin family, namely epigallocatechin gallate (EGCG). Gallic acid was in a value of (2125.1 µg/g), which is a derivative of hydroxybenzoic acid, followed by 977.7 µg/g of acid chlorogenic acid. Moderate Levels of Phenolics (100–700 µg/g). The caffeine content (740.9 µg/g), elagic acid content of 494.3% per gm and 412.8 µg of rutin per gm. Phenols with somewhat higher potencies (10–100 µg/g) includes Pyrocatechol (82.8 µg/g) and syringic acid (61.4 µg/g), Daidzein (50.4 µg/g) and quercetin (41.1 µg/g). Phenolics below 50 µg/g, or trace levels includes Rosmarinic Acid (27.5 µg/g), Coumaric Acid (1.8 µg/g), Kaempferol (14.5 µg/g), Hesperetin (5.3 µg/g), Vanillin (14 µg/g), and Cinnamic Acid (2.7 µg/g). These phenolics are only present in trace amounts.

**Table 1:** Phenolic compounds in the green tea beverage detected by HPLC analysis.

Phenolics by HPLC	Green tea (µg/g)
Catechin	70190.8
Gallic acid	2125.1
Chlorogenic acid	977.7
Coffeic acid	740.9
Ellagic acid	494.3
Rutin	412.8
Pyro catechol	82.8
Syringic acid	61.4
Daidzein	50.4
Querectin	41.1
Rosmarinic acid	27.5
Ferulic acid	16
Kaempferol	14.5
Vanillin	14
Naringenin	11
Hesperetin	5.3
Cinnamic acid	2.7
Coumaric acid	1.8
Methyl gallate	0

### Albendazole resistance detection

Fecal examination revealed that sheep were infected with strongyle group, larval identification showed that *H. contortus* larvae was the most infective larvae (113/150) 75.3% followed by *Trichostrongylus* spp. (22/150) 14.66% and *Ostertagia* spp. (15/150) 10%. The highest hatch inhibition percentage was detected at an albendazole concentration of 25 µg/mL. Meanwhile, the lowest hatch inhibition percentage was detected at a concentration of 0.012 µg/mL. By decreasing the albendazole concentration, a significant decrease in mean unhatched egg count and the mean egg hatch inhibition percentage (*p* ≤ 0.0001) was observed (Table 2). Egg hatch inhibition test exposed occurrence of albendazole resistance among Strongyle group with high percentage of *H. contortus*, whereas LC_50_ of albendazole was at concentration 0.172 µg/mL.

**Table 2:** Mean number of un-hatched strongyle group eggs recovered from naturally infected sheep, and hatching inhibition % post treatment with different albendazole concentration.

Albendazole conc. µg/mL	25	12.5	6.25	3.125	1.56	0.78	0.39	0.195	0.097	0.048	0.024	0.012	Control
Mean number of un-hatched eggs	89.3 ± 2.9^a^	86.3 ± 3.2^ab^	84.3 ± 2.3^ab^	79.7 ± 2.7^bc^	78.3 ± 4^bc^	73. 7 ± 4^cd^	68. 7 ± 3.7^d^	60 ± 0^e^	53. 7 ± 1.3^e^	37 ± 1.5^f^	31. 7 ± 1.6f^g^	26. 7 ± 2.9^g^	9.3 ± 3.^h^
Hatching Inhibition%	88.23	84.92	82.72	77.57	76.10	70.95	65.44	55.88	48.89	30.51	24.63	19.11	0

Different small letters indicate significance between different concentrations to control.

### Anthelmintic efficacy against resistant *H. contortus* isolate

#### The inhibitory effects of the GT against eggs and larvae

**Fig. 2:**
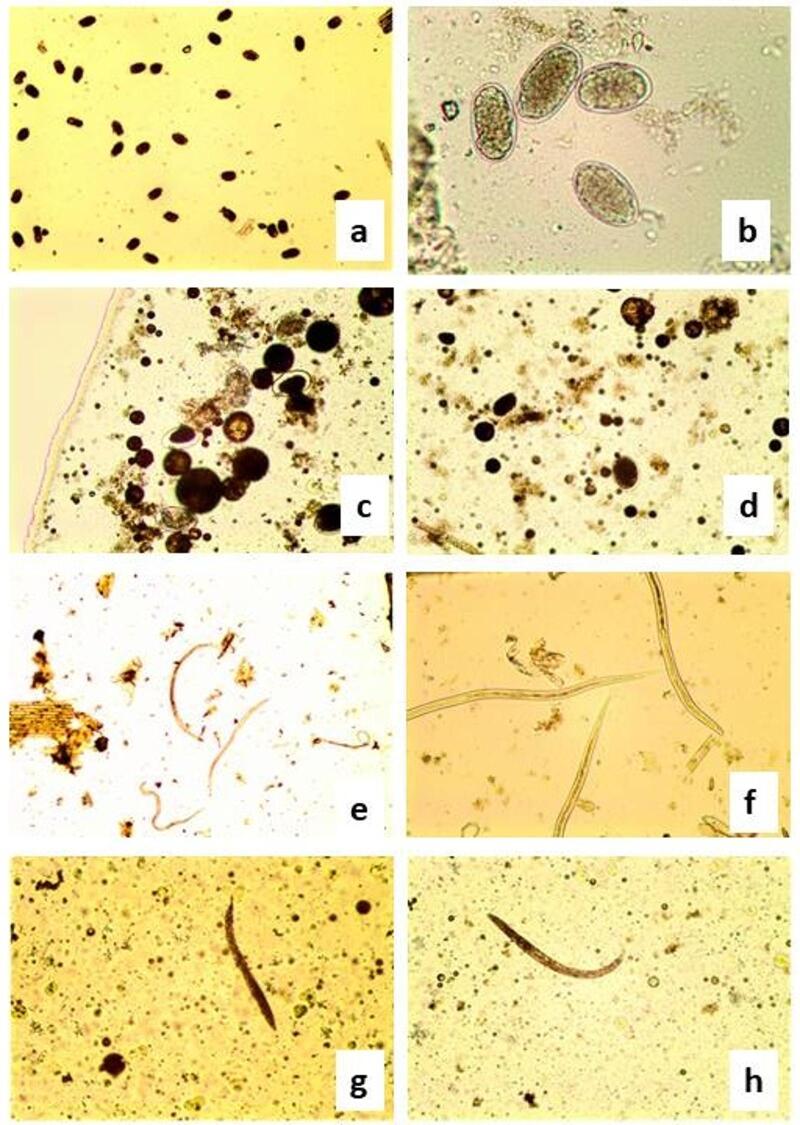
Structural deformities of *H. contortus* eggs and larvae exposed to GT compared to the normal ones. a and b; *H. contortus* eggs with grayish thin wall containing morula stage showing normal cleavages of egg cells (4×) and (20×), respectively. c and d; GT treated *H. contortus* eggs showing shrinkage, darkness and absence of normal appearance of egg cells cleavages (10×). e; Normal *H. contortus* larvae (10×), f; Anterior rounded end and posterior tapered tail of *H. contortus* larvae (20×), g and h; GT treated larvae showing absence of the normal appearance with deformities (10×).

The GT exhibited a great inhibitory egg hatching activity against *H. contortus* eggs (Table 3). By decreasing the concentration of the extract, the inhibitory effect of GT decreased. The extract resulted in 100% inhibition of egg hatchability at high concentrations (50 and 25 mg/mL) (р<0.001). Superior inhibitory effects were also observed 98.9% to 94.5% at (12.5 and 6.25 mg/mL), respectively. The lowest egg inhibition effect of GT (41%) was noted at the concentration of 0.048 mg/mL. Where un-hatched egg count was recorded (41.0 ± 6.8), which was significant in comparison to control (8.3 ± 2). The GT lethal concentration (LC_50_) was 0.144 mg/mL, while lethal concentration (LC_90_) was 3.841 mg/mL. Table 3 showed a significant motility inhibition activity on *H. contortus* larvae at the high concentration of GT (50 and 25 mg/mL), whereas it reached 99% and 95%, respectively. There was concentration dependence efficacy of the extract on larvae. The lowest GT concentration (0.048 mg/mL) caused superior anti larval effect where the mean count of non-motile larvae was 40.3 ± in comparison to the control (5.7 ± 0.88). The LC_50_ was 0.127 mg/mL (upper limit 0.08, and lower limit 0.18515), while LC_90_ was 12.124 mg/mL (upper limit 7.66944 and lower limit 21.77892). Varies structural deformities of *H. contortus* eggs and larvae exposed to GT compared to the normal ones were illustrated in Fig. 2a–h. Normal *H. contortus* eggs appeared with grayish thin wall containing morula stage showing normal cleavages of egg cells (a and b). GT treated *H. contortus* eggs showed shrinkage, darkness and absence of normal appearance of egg cells cleavages (c and d). No changes were found on *H. contortus* larvae before treatment where the larvae showed normal anterior rounded end (e and f) and posterior tapered tail end. GT treated larvae showed absence of the normal appearance with significant deformities (g and h).

**Table 3:** Mean count ± SE of un- hatched eggs, and non motile larvae post exposure to different GT concentrations in comparison to control untreated ones.

GTE Concentrations	Mean un-hatched egg count ± SE Post 24 h	Mean non motile Larva count NTl ± SE Post 48 h
50 mg/mL	100 ± 0^a^	99.7 ± 0.25^a^
25 mg/mL	100 ± 0^a^	95.67 ± 4.3 ^a^
12.5 mg/mL	99 ± 1^a^	91 ± 1 ^a^
6.25 mg/mL	95 ± 2.8^a^	81 ± 3.7^b^
3.125 mg/mL	88.3 ± 6^ab^	78.3 ± 2.4^bc^
1.56 mg/mL	80.3 ± 5.7a^bc^	76 ± 2 ^bc^
0.78 mg/mL	73. 7 ± 6.9^bc^	69.3 ± 2.9^cd^
0.39 mg/mL	66.0 ± 10.6^cd^	64. 7 ± 3.2^de^
0.19 mg/mL	62. 7 ± 9.9^cd^	60 ± 2.6^e^
0.097 mg/mL	49.0 ± 6.6^de^	49. 7 ± 3.5^f^
0.048 mg/mL	41.0 ± 6.8^e^	40.3 ± 5^g^
Control untreated	8.3 ± 2^f^	5. 7 ± 0.88^h^
P-value	0.000	0.000
F-value	82.913	78.792

Different small letters indicate significance between concentration (column).

### Adult motility inhibition activity

Various concentrations of the GT exhibited anthelmintic potency on the adult worms in comparison with the control ones (*p* < 0.001) (Table 4). The anti-nematodal action of the GT was concentration and time dependent. The concentration of 400 mg/mL post 2 h caused the most effective motility inhibition effect (100%). Similar superior effects were recorded at concentration of (100 and 200 mg/mL) after (4 h and 6 h), respectively. The LC_50_ was 13.387 mg/mL, while LC_90_ was 59.105 mg/mL. The complete immobile worms considered dead ones. Worm motility inhibition %, and mortality index post 24 h treatment was (100% and 1), respectively in the highest concentrations (100 to 400) mg/mL. However, they declined by decreasing their concentration to reach (28.2% and 0.415), respectively at 6.25 mg/mL.

**Table 4:** Mean number of immotile worms ± standard errors following adult worm treatment with different the GT concentrations during varies interval time of exposure.

GTE concentration	Mean number of immotile worms ± SE				WMI%	MI
	2 h	4 h	6 h	24 h		
400 mg/mL	100 ± 0.0^a^	100 ± 0.0^a^	100 ± 0.0^a^	100 ± 0.0^a^	100%	1
200 mg/mL	75 ± 2.8^b^	100 ± 0.0^a^	100 ± 0.0^a^	100 ± 0.0^a^	100%	1
100 mg/mL	58.3 ± 1.7^c^	76. 7 ± 6^b^	100 ± 0.0^a^	100 ± 0.0^a^	100%	1
50 mg/mL	45 ± 2.8^d^	61. 7 ± 1.7^c^	76. 7 ± 1.7^b^	88.3 ± 6^b^	85.8%	0.8
25 mg/mL	30 ± 2.8^e^	43.3 ± 1.7^d^	56. 7 ± 1.7^c^	63.3 ± 3.3^c^	55.2%	0.635
12.5 mg/mL	21. 7 ± 1.7^f^	36. 7 ± 1.7^de^	46. 7 ± 1.7^d^	50 ± 2.8^d^	38.6%	0.05
6.5 mg/mL	18.3 ± 1.7^f^	30 ± 0.0^f^	36. 7 ± 1.7^e^	41. 7 ± 1.7^e^	28.2%	0.415
Control (DW)	0.0 ± 0.0^g^	5 ± 2.8^g^	10 ± 0.0^f^	18.3 ± 1.7^f^		
P-value	0.000	0.000	0.000	0.000		
F-value	260.274	176.241	826.857	130.019		

Different small letters indicate significance between concentrations (column).

#### Alterations in cuticle of adult worms

##### Light microscopic findings

**Fig. 3:**
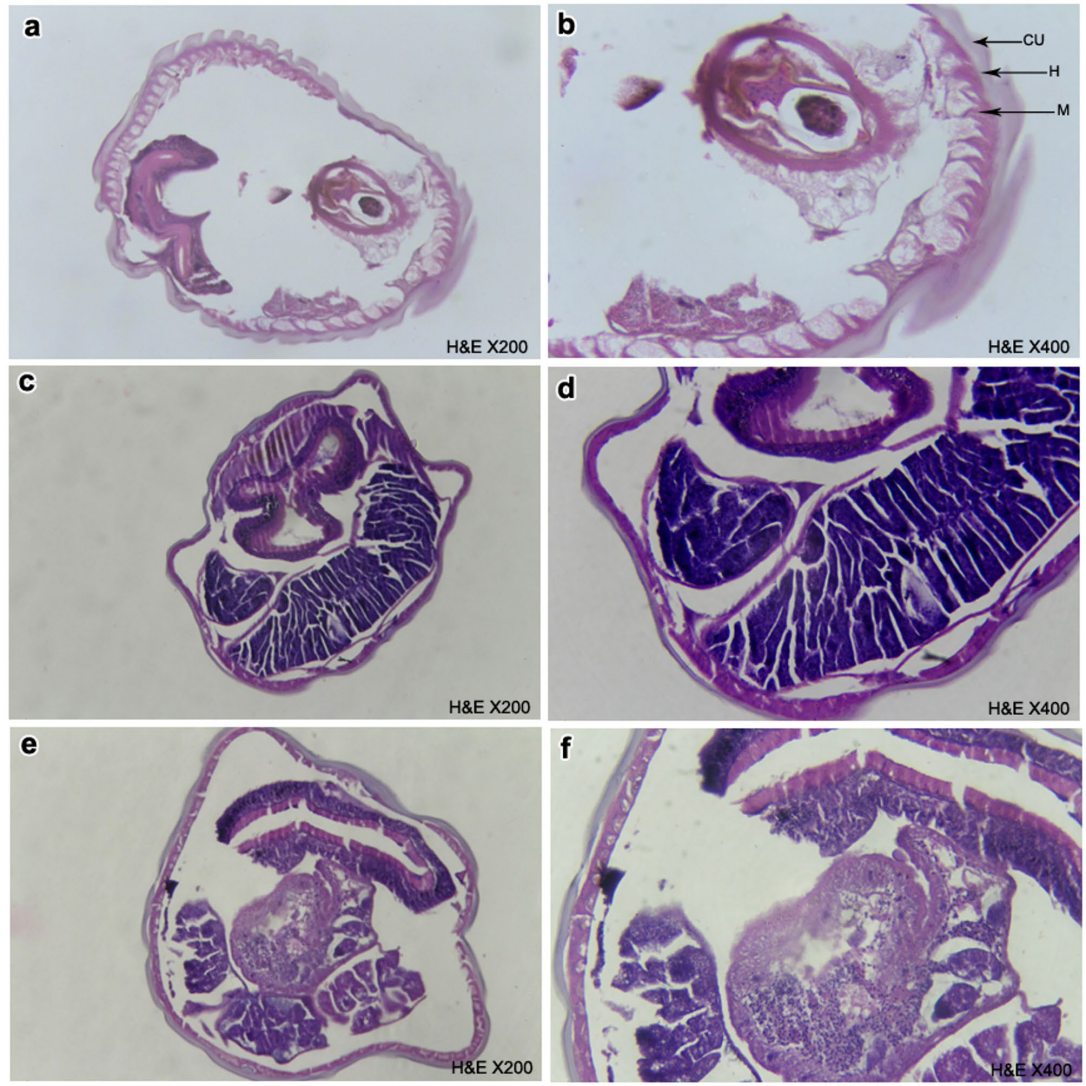
Light microscopy of cut-section of adult *H. contortus* worm post exposure to GT for 6 h and 24 h compared to control untreated ones. (a and b) control untreated, (c and d) 6 h GT post exposure and (e and f) 12 h GT post exposure.

The cuticle of untreated *H. contortus* adult worm (Fig. 3a and b) showing three layers including outer cuticle, followed by thin hypodermis which comprised of a syncytium of cells. Finally, several layers of longitudinally arranged striated muscles suited internally. The adult worm incubated for 6 h at concentration of 400 mg/mL the GT (Fig. 3c and d) showed alteration in the integrity of the cuticle which appeared to be thinner. The inner muscle layer of cuticle lost its normal appearance showing disarrangements. Areas of joining discontinuity between the cuticle and the muscle layer could be observed. By increasing of 400 mg/mL the GT exposure time for 24 h (Fig. 3e and f) severe distortion of cuticle wall was more remarkable, whereas large areas of cleavage and degeneration were detectable in the muscular layer of cuticle.

#### Scanning electroscopic observation

**Fig. 4:**
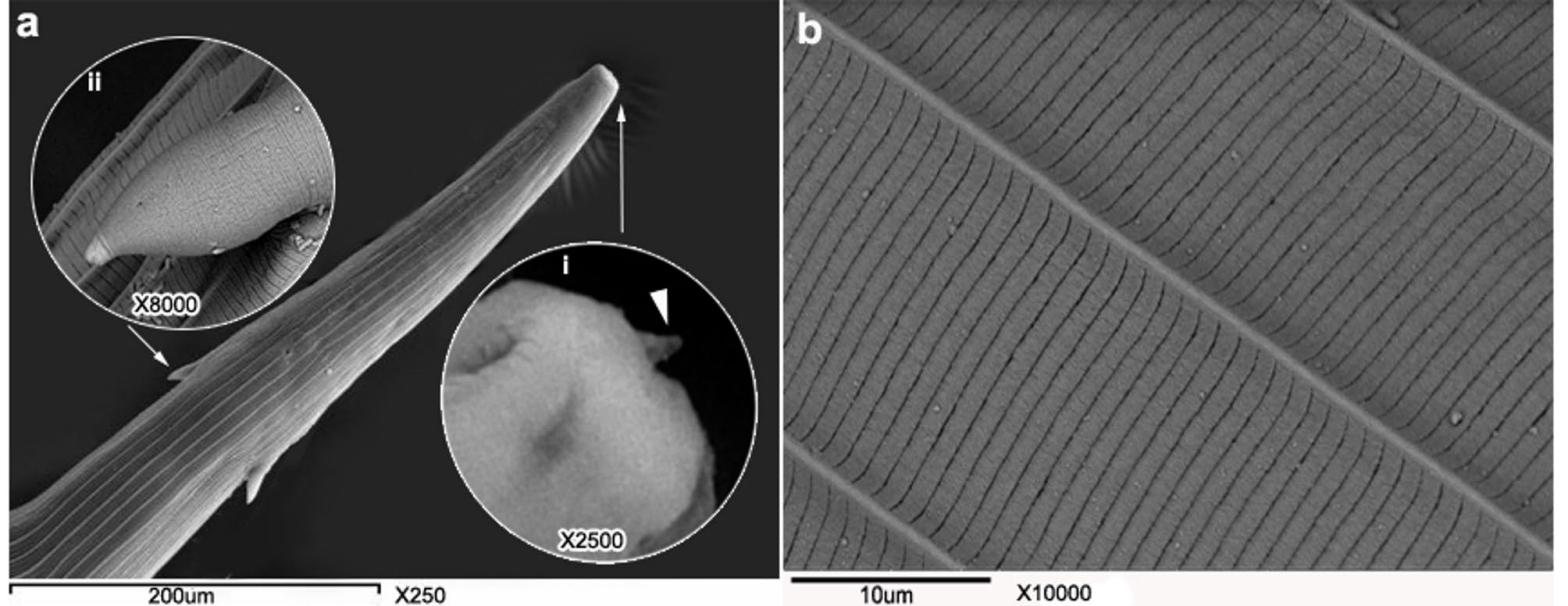
SEM of control untreated *H. contortus* adult worm showing normal buckle capsule, cervical papillae (**a**) and cuticle surface aspect (**b**). (i: dorsal lancet tip, ii: cervical paillae).

The mouth of untreated adult worm was hexagonal containing semicircular rudimentary lips, lateral amphids, and papillae with detectable dorsal buccal lancet. The lancet’s anterior tip and lateral edges were slightly rounded. Two spine-like cervical papillae were also observed. The cuticle has transverse striation formally suited and remarkable lateral ridges as shown in Fig. 4a and b.

**Fig. 5:**
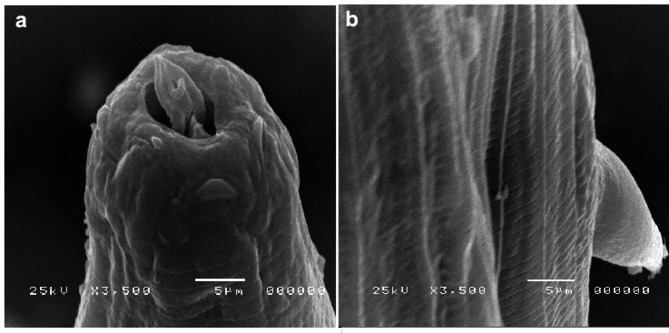
SEM of *H. contortus* adult worm buccal capsule (**a**) and cuticle surface and cervical papillae (**b**), post exposure of 400 mg/mL GT showing severe degrees of distortion.

Severe distortion and damages in both buccal capsule and the entire cuticle of adult worm, following 24 h treatment with 400 mg/mL the GT were observed as shown in Fig. 5a and b. The lips and dorsal lancet tip exposed deformity and oedema. The cuticular surface and cervical papillae appeared to be enlarged. Moreover, the transverse striation of cuticle lost the normal appearance. The longitudinal ridges of cuticles were being fainted and corrugated with prominent wrinkling.

### Cytotoxic assessment

**Fig. 6:**
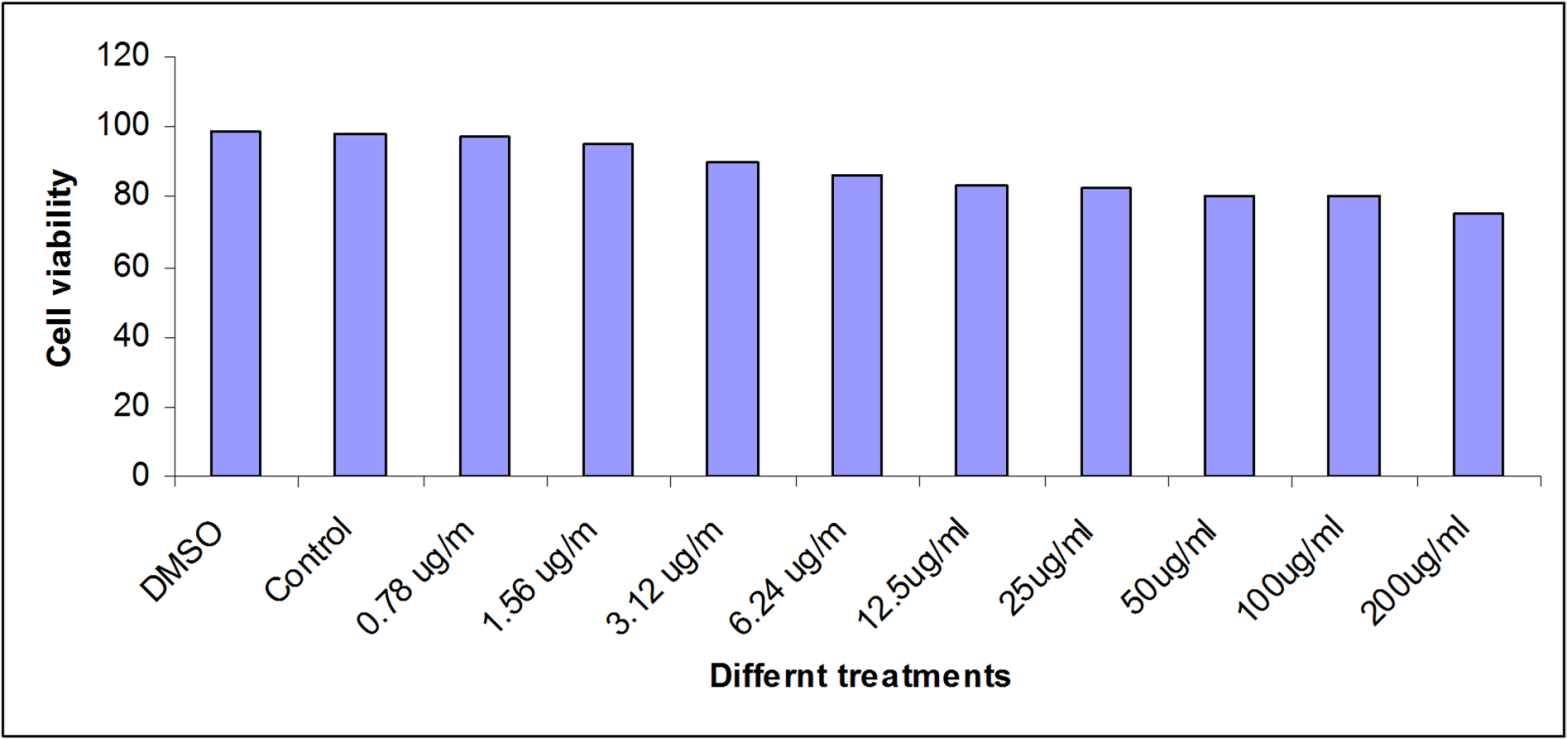
Cell viability of BJ1 cells post exposure to varies concentrations of the GT, DMSO and Control.

The cytotoxic effects of the GT on the viability of the BJ1 cell lines using MTT assay are illustrated as percent cell viability in Fig. 6. The MTT assays revealed that cell viability decreased by increasing concentration. The GTE at different concentrations (0.78 µg/mL to 200 µg/mL) had no cytotoxic effects compared to those of (100%, 1% and 2%) for the positive control, DMSO and control negative, respectively.

## Discussion

*H. contortus* remains the main multidrug resistant strongyle threatened livestock productivity all over the world^4,45^. The present coprological examination revealed the mass spread of strongyles group among the examined sheep, where *H. contortus* larvae was the most infective larvae 75.3%. This goes parallel with the high incidence of *H. contortus*infection among livestock in various Egyptian provinces, recorded by Osman et al.^46^, in New-Valley, Hassan et al.^47^, in Beni-Suif, Hassan et al.^3^, in Giza, Mohamed et al.^48^, in Upper Egypt. This extreme spread might be attributed to the direct life cycle criteria of this nematode, immunity and nutrition status of animal side as well as, the suitable climatic condition of the semi-temperate region of Egypt. Anthelmintic resistance is one of the substantial threats of the livestock productivity causing enormous economic losses all over the world^8^. The current study evoked evidence of albendazole resistance among *H. contortus* population that had been cleared through egg hatch inhibition test where LC_50_ of albendazole was at concentration 0.172 µg/mL which was found to be more than the limited value 0.1 µg/mL^28^. This anthelmintic resistance might be due to misuse of albendazole known as one of the most common anti-parasitic commercial chemotherapeutics all over the world^8^. In other context, Aboelhadid et al.^9^, reported albendazole and ivermectine resistance among sheep naturally infected in Beni-Suef province, Egypt. Furthermore, Hassan et al.^4^, found that *H. contortus* was the main prevalent species infecting sheep, despite using of albendazole as anti-parasitic agent, in Giza and Cairo provinces, Egypt. Therefore, emerging eco-friendly beverage can combat resistant *H. contortus* isolations, either by alternating the existing artificial therapeutic or strengthening its effect is a need.

Green tea is one of the precious plants containing great biological constituents of remarkable health benefits^49,50^. Characterization of GT bioactive compounds exhibited presence of high total polyphenolic component content of green tea. The green tea beverage demonstrated an exceptionally strong capacity to neutralize free radicals. Green tea’s antioxidant capacity is known to be influenced by polyphenols, especially catechins (like EGCG). Several tests, including Total Phenolic Content (TPC), DPPH radical scavenging activity, DPPH antioxidant activity as vitamin C equivalents, and Ferric Reducing Antioxidant Power (FRAP), revealed that the examined green tea samples continuously exhibited strong antioxidant qualities. The ability of green tea to fight oxidative stress is supported by a scavenging activity exceeding 80%, which is typically regarded as outstanding. These findings provided more evidence for the link between effective radical scavenging and a high phenolic content^51^. Green tea extract displayed valuable vitamin C equivalent. This suggests that it may contain molecules with significant redox potential. In the same context, the ability of the extract to convert Fe³ to Fe², which is correlated with electron-donating antioxidant activity, was in line with high total phenolic contents, which lends further credence to the theory that polyphenols constitute the main source of antioxidant activity. The high polyphenolic contents of gallic acid and catechins beside the unique antioxidant activity of green tea extract were also previously reported by^52–56^. The HPLC study of green tea revealed a rich and diverse profile of phenolic compounds, but with significant variance in their quantities^57,58^. Green tea’s potent anti-inflammatory, antioxidant, and health-promoting properties are mostly due to these compounds^59,60^. Catechin was the most common phenolic component found in green tea. The epigallocatechin gallate (EGCG); the important flavan-3-ol belongs to the catechin family, possesses antioxidant, cardiovascular, and anti-cancer activities^49,61^. The high concentration catechins are primarily responsible for the significant antioxidant capacity shown in DPPH and FRAP tests. Furthermore, gallic acid, which is a derivative of hydroxybenzoic acid possesses antioxidant, antimicrobial, and anti-inflammatory qualities. It could also enhance catechins’ ability to scavenge radicals by cooperating with them^62^. Chlorogenic acid and caffeic acid have antioxidants and anti-diabetic qualities. This improves the system’s capacity to scavenge free radicals, as evidenced by its moderate concentration. The high caffeine content of GT (740.9 µg/g) in addition to its well-known anti-inflammatory and antioxidant qualities, caffeine may also offer heart-healthy benefits^63^. Moreover, ellagic acid content of 494.3% per gm reported to have an anticarcinogenic and hepatoprotective dimeric derivative of gallic acid, more over its contents of flavonoid glycoside 412.8 µg of rutin per gm which has strengthen role to blood capillaries and reduces inflammation^64^.

HPLC demonstrated that GT contained Pyrocatechol and syringic acid that promote antioxidant and antibacterial properties. Moreover, it had Daidzein and quercetin which are isoflavones and flavonols with oestrogenic and anti-inflammatory properties. Their presence enhances the functional profile of green tea, especially in hormone-related health areas. Other phenolics with trace levels were Rosmarinic acid, Coumaric acid, Kaempferol, Hesperetin, Vanillin, and Cinnamic acid, which had potent bioactivity. The HPLC analysis confirmed the abundance of substantial bioactive contents of green tea beverage that previously recorded by Robertson and Bendall^65^; Maslov et al.^66^; Harfoush et al.^56^.

Multiple studies recognized catechins derived from green tea as promising anthelminthic compounds affecting on biotic activity of helminths^50^. The current anthelmintic efficacy of the GT has been proven by its substantial inhibitory activity against eggs, larvae and adults of the resistant *H. contortus* isolates. Different concentrations of the GT exhibited anthelmintic potency, and there was concentration dependence efficacy of the extract on this resistant gastric nematode. Significant egg hatch inhibition (100%) and larval motility inhibition (99 and 95%), % were detected at high concentrations of (50 and 25 mg/mL), respectively. Severe deformities on *H. contortus*eggs and larvae were observed. Adulticidal effect of the GT through promoting paralysis or even death of adults was obvious. The concentration of 400 mg/mL post 2 h caused the most effective motility inhibition effect (100%). Worm motility inhibition % and Mobility index post 24 h treatment was (100% and 1), respectively in the highest concentrations (100 to 400) mg/mL. These results coincide with multiples of studies mentioned the anti-parasitic effect of green tea. Zhong et al.^26^, recorded the ability of the dietary green tea polyphenols supplementations for infected lambs to reduce adult *H. contortus*burden. Karonen et al.^67^, revealed the inhibitory effect of ellagitannins derived from green tea on the exsheathment of third-stage infective larvae of *H. contortus* and *T. colubriformis*through the in vitro larval exsheathment inhibition assay. Ramdani et al.^27^, found that addition of green tea dust; a by-product of green tea fabrication to ruminant diets had potential anthelmintic activity against strongyle worms owing to its high protein and bioactive contents. The anti-larval activity of green tea might be related to its content of phenolic tannins which in turn could bind to the free protein and reduced the larval nutrient availability, causing larvae starvation and death^68^. As well as Argüello-García and Quiñonez-Bastidas^50^ claimed the potent impact of catechins on motility and mortality of helminths.

In other context, the various constitutes of the green tea proved the anti-parasitic potency with protozocidal action^69^. Moreover, Aboulaila et al.^70^, claimed that green tea could cause growth inhibition for *Babesia bovis* and *Babesia bigemina*. Meanwhile it prompted production of intracellular H_2_O_2_ and depolarization of the mitochondrial membrane of *Leishamania amazonenesis*^24^. It is also the responsible of inhibition of arginine kinase in *Plasmodium berghei*^23^. Fakae et al.^71^, reported anti-*acanthamoebic* activity of green tea hot and cold brews against trophozoite and cystic forms of *A. castellanii*. Fakae et al.^25^, referred to the remarkable cysticidal effect of *C. sinensis* solvent extracts through *A. castellanii* trophozoite replication inhibition effect, which might be related to two constituents of *C. sinensis*, namely epigallocatechin-3-gallate and caffeine.

This study also proved that the GT caused severe distortion of cuticle wall of the adult *H. contortus*, with large areas of cleavages and degeneration in the muscular layer of cuticle. This might be agreed with Fakae et al.^71^, studied the structural alterations in *C. sinensis*-treated trophozoites of *Acanthamoeba castellanii*utilizing transmission electron microscopy and scanning electron microscopy. And stated its destructive effect on the cell membrane’s integrity trophozoites and caused progressive damage of trophozoites in a dose-dependent manner. Moreover, Julaeha et al.^68^, who stated that condensed tannins might join to the cuticle of parasite, which is high in glycoprotein. Bioactive compounds e.g. alkaloids, flavonoids, saponins, and phenolic tannins could move into the body of parasite by the transcuticular diffusion process, thus the energy formation process and absorption of nutrients is harmed. So, death of larvae might occur as because of larval development inhibition as a result of the reduced amount of energy and electrolytes^68^.

The MTT colorimetric assay assigned the functional state of mitochondria, monitoring cell viability. A mitochondrial dehydrogenase enzyme in living cells reduces yellow tetrazolium MTT salt to blue MTT formazan, which is settled in undamaged cells^72^. The prepared GTE exhibited no cytotoxic effect on BJ1 cells. This is similar to the findings of Fakae et al.^71^, who recorded low cytotoxic activity of *C. sinensis* on primary corneal stromal cells. In vitro maintaining mammalian cells in health status needs special factors like serum growth factors, and other nutrients in the culture medium, which are required for normal physiology, metabolism and viability of cultured cells^69,73^. So, the prepared GT has maintained cell viability, and this has proved that cultured BJ1 cells have gained all the nutrition required for their viability. Therefore, it could be safe to use GT as oral treatment for live animals against parasitic infection or as nutritional supplements that promote health.

## Conclusion

It is concluded that *H. contortus* is the most prevalent *Strongyle* infecting Egyptian sheep. The study alerts from high exceeding existence of resistance to albendazole among population of *H. contortus* in Egypt. Green tea beverages are affluent of biomedical phyto-components; catechin, gallic acid, caffeine, ellagic acid and rutin as the dominant phenolics, with possible combined synergistic effect, in exerting substantial antioxidant ability and brilliant biocidal activity against *H. contortus* isolate resistant to albendazole. This study highly recommended the green tea beverage as safe, potent antioxidant and phyto-therapeutic, alternative to chemotherapeutics, that may control anthelmintic inefficiency particularly for the resistant parasites.

## Data Availability

All data obtained is included in this manuscript and is available on request from the corresponding author.
